# Early Donor-Specific HLA Antibodies Detected by Screening in the First Month Posttransplant and Kidney Graft Outcomes

**DOI:** 10.3389/ti.2025.14424

**Published:** 2025-08-06

**Authors:** Covadonga López del Moral, David San Segundo, María José Ortega, Miguel Martínez-Belotto, Rosalía Valero, Lara Belmar, María de la Oliva Valentín, Emilio Rodrigo, Marcos López-Hoyos, Juan Carlos Ruiz

**Affiliations:** ^1^ Department of Nephrology, Marqués de Valdecilla University Hospital-IDIVAL, Santander, Spain; ^2^ Department of Immunology, Marqués de Valdecilla University Hospital-IDIVAL, Santander, Spain

**Keywords:** kidney transplant, antibody-mediated rejection, donor-specific antibodies, graft outcomes, HLA screening

## Abstract

Donor-specific HLA antibodies (DSA) are related to antibody-mediated rejection (ABMR) and graft failure. The rationale and frequency of screening for anti-HLA antibodies in stable patients are not established. The aim of our study is to evaluate the impact of early DSA appearance in the first month post-transplant on graft outcomes. All kidney transplant recipients between 1/1/2012–12/31/2022 with anti-HLA antibody screening by Luminex during the first month post-transplant were included. Patients with preformed or historical DSA and those with DSA detection after graft loss were excluded. The mean fluorescence intensity cut-off was 1,500. Three hundred fifty-three patients were included and the median time from transplant to first antibody sample was 30.0 days. During 3.8 years of follow-up, graft loss occurred in 9.1% and 19.5% had ABMR. A total of 8.5% developed early-DSA in the first month. Patients with early-DSA detection had more HLA sensitization at the time of transplant (p < 0.001). Multivariable analysis showed that the presence of early-DSA was an independent risk factor for ABMR. In conclusion, sensitized patients at the time of transplant have a higher risk of DSA formation in the first month, probably reflecting alloimmune memory, therefore early HLA antibody screening should be performed in this high-risk population.

## Graphical Abstract

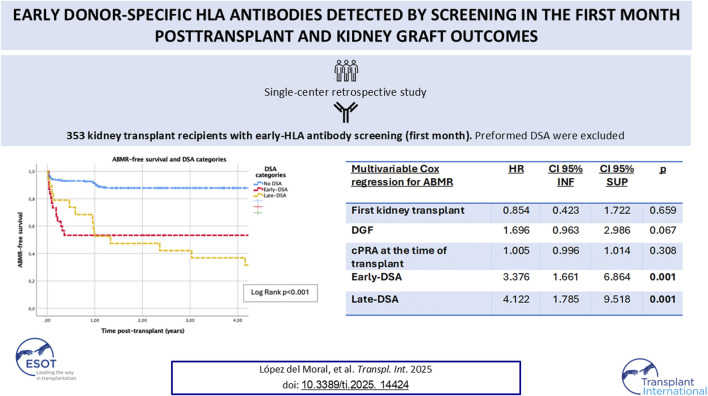

## Introduction

Donor-specific HLA antibodies (DSA) are a key factor for the diagnosis of antibody-mediated rejection (ABMR) and are associated with poor outcomes after kidney transplantation [1–6]. Immunological risk assessment before and after transplant has improved with solid-phase immunoassays in the Luminex system that provide sensitive and specific information on HLA antibodies with screening and single-antigen bead (SAB) assays, but their results must be interpreted appropriately [7–14]. In particular, the semiquantitative value of mean fluorescence intensity (MFI) and the problem of establishing a fixed and universal MFI positivity threshold hinder the correlation of DSA with clinical outcomes and the unification of results [15, 16].

Preformed and *de novo* DSA (dnDSA) are related to alloimmune injury [17–19]. The risk factors for dnDSA development are, among others, under-immunosuppression, graft inflammation and high HLA mismatch [17, 20–24]. Although dnDSA may appear at any time after transplantation, the STAR Working Group notes that the development of DSA between 2 weeks and 3 months post-transplant may represent a memory response [15, 25]. A history of HLA sensitizing events such as previous transplants, pregnancies or blood transfusions, and the presence of non-DSA HLA antibodies prior to transplant are risk factors for latent alloimmune memory [15].

Despite the impact of DSA on graft outcomes, there is still no consensus on the indication for DSA screening after transplantation in stable patients. Recently, the OuTSMART trial demonstrated that optimization of baseline immunosuppression after DSA detection had no impact on graft survival [26], showing that universal screening may be controversial, and the cost-effectiveness of this strategy is not determined [27, 28]. Furthermore, the frequency of routine surveillance for HLA antibodies is not established [15], and a recent ESOT Working Group proposed a monitoring scheme with screening in the first 3–6 months after transplant and annually thereafter (2C recommendation) [29]. Although an earlier assessment of HLA antibodies has been suggested in patients at potential risk of latent memory [15, 30], the exact timing of the first post-transplant HLA determination is not currently settled. Moreover, most series have described early DSA as those detected in the first year posttransplant [31–35], and there are few data on the specific evaluation of HLA monitoring in the first month.

The aim of our study is to evaluate the impact of early HLA antibody screening in the first month posttransplant on kidney graft outcomes and identify patients at risk of early DSA formation.

## Materials and Methods

### Study Population

For this retrospective analysis we included all kidney transplant recipients from 1/1/2012–12/31/2022 at Marqués de Valdecilla University Hospital with HLA antibody screening during the first month post-transplant (range 10–60 days). Those patients with preformed or historical DSA described in pre-transplant sera and with positive flow cytometry crossmatch (FCXM) were excluded. Patients with graft failure in the first 60 days and those who developed DSA after graft loss were also excluded. The primary outcome variable was time to antibody-mediated rejection. The study was conducted according to the guidelines of the Declaration of Helsinki and was approved by the regional Ethics Committee of our institution (2024.196).

Demographic and clinical data, including recipient and donor data (type, age), induction immunosuppression, cold ischemia time (CIT), delayed graft function (DGF), HLA mismatch, early blood transfusions - within the first 30 days after transplant, and biopsy data were collected from the prospectively maintained database of renal transplant patients at our center. All rejections were categorized according to Banff 2019 classification [36]. Allograft biopsies were performed by clinical indication and by protocol 1-year after transplantation.

Our induction protocol consisted of anti-thymocyte globulin (ATG) in highly sensitized patients and in some patients with a previous transplant lost due to rejection. Anti-IL2R was administered in patients at high risk of post-transplant acute tubular necrosis, primarily due to advanced donor age, prolonged expected CIT, or donation after circulatory death. The corticosteroid treatment protocol at our center was an intravenous pulse of 500 milligrams (mg) of methylprednisolone for induction. Oral prednisone was continued at 20 mg for the first 2 weeks after transplant, 15 mg of prednisone 2 weeks later until the first month and 10 mg at 1 month after transplant, with a subsequent reduction to 7.5 mg after 2 months and 5 mg after 3 months. Discontinuation or maintenance of baseline prednisone after 3 months was performed individually and according to clinical indication.

### HLA Antibodies

Regular monitoring of HLA antibodies was performed in the first- and sixth-month post-transplant and annually thereafter as routine clinical practice in our center, and in case of signs of impaired allograft function or clinical request. Patients had pre- and post-transplant sera screened using a mixed panel beads (LABScreen Mixed Class I and II, One Lambda, Canoga Park, CA) and if a positive result was detected, further LABScreen^®^ SAB assay class-I and class-II (One Lambda, Canoga Park, CA) was performed by Luminex^®^ technology. Pre-transplant sera before 2012 were assessed by enzyme-linked immunosorbent assay (ELISA) as this was the available technique, but all patients had at least one pre-transplant serum screened by Luminex^®^. According to the policy of our center, anti-HLA antibody testing was performed every 3 months in patients on the transplant waiting list. An additional anti-HLA antibody sample was collected on day 0 if sensitizing events, such as blood transfusions, occurred between the day of transplant and the last serum sample. The last pretransplant anti-HLA antibody sample was used to calculate pretransplant cPRA, but all pretransplant sera were reviewed to exclude patients with preformed or historical DSA.

The general positivity threshold in our laboratory was set at 1,500 MFI, and the presence of DSA was defined by the Histocompatibility laboratory considering the MFI positivity cut-off and other factors such as the evolution of HLA antibodies posttransplant or epitope sharing phenomena [15, 16]. In laboratory routine, we included dilution sera in highly sensitized patients and in those with suspected prozone, as described [37].The most probable 2-field HLA typing of the donor [38] and haplotype frequencies [39, 40] for missing information on specific HLA loci were considered to assign DSA. Calculated panel-reactive antibody (cPRA) was obtained through the Virtual PRA Calculator of the Eurotransplant Reference Laboratory [41], and delta cPRA >0% was recorded (difference between cPRA in the first serum at 1-month post-transplant and cPRA in the last pre-transplant serum).

### Patient Groups

Patients with early DSA detection in the first month (10–60 days) posttransplant were categorized as “early-DSA,” and those patients with first DSA detection >60 days - and without DSA in the first month - were categorized as “late-DSA.” Patients without DSA detection during the follow-up period were classified as “no-DSA.” “Transient” DSA was defined as disappearance of DSA at 3 and/or 6 months after first detection (if >1 DSA per patient, disappearance of at least one DSA).

### Statistical Analysis

Continuous variables were expressed as mean ± standard deviation (SD) or median and interquartile range (IQR) according to their distribution. Categorical variables were described as relative frequencies. Continuous variables with non-normal distribution were compared using non-parametric tests (Mann-Whitney U test to compare 2 groups and Kruskal-Wallis test to compare 3 groups). A chi-square test was used to compare the average values of categorical variables. Univariable and multivariable Cox regression were performed to determine which variables were associated with ABMR, and hazard ratios (HR) were reported with 95% confidence intervals. To evaluate the predictive capacity of DSA by Cox regression, patients with ABMR before DSA appearance were eliminated from this analysis. Time-to-event outcome data were assessed by Kaplan–Meier plots and log-rank tests. A p-value <0.05 was defined as statistical significance. Statistical analysis was conducted using the SPSS statistical software package (Version 25.0. Armonk, NY: IBM Corp.).

## Results

### Baseline Characteristics

**FIGURE 1:**
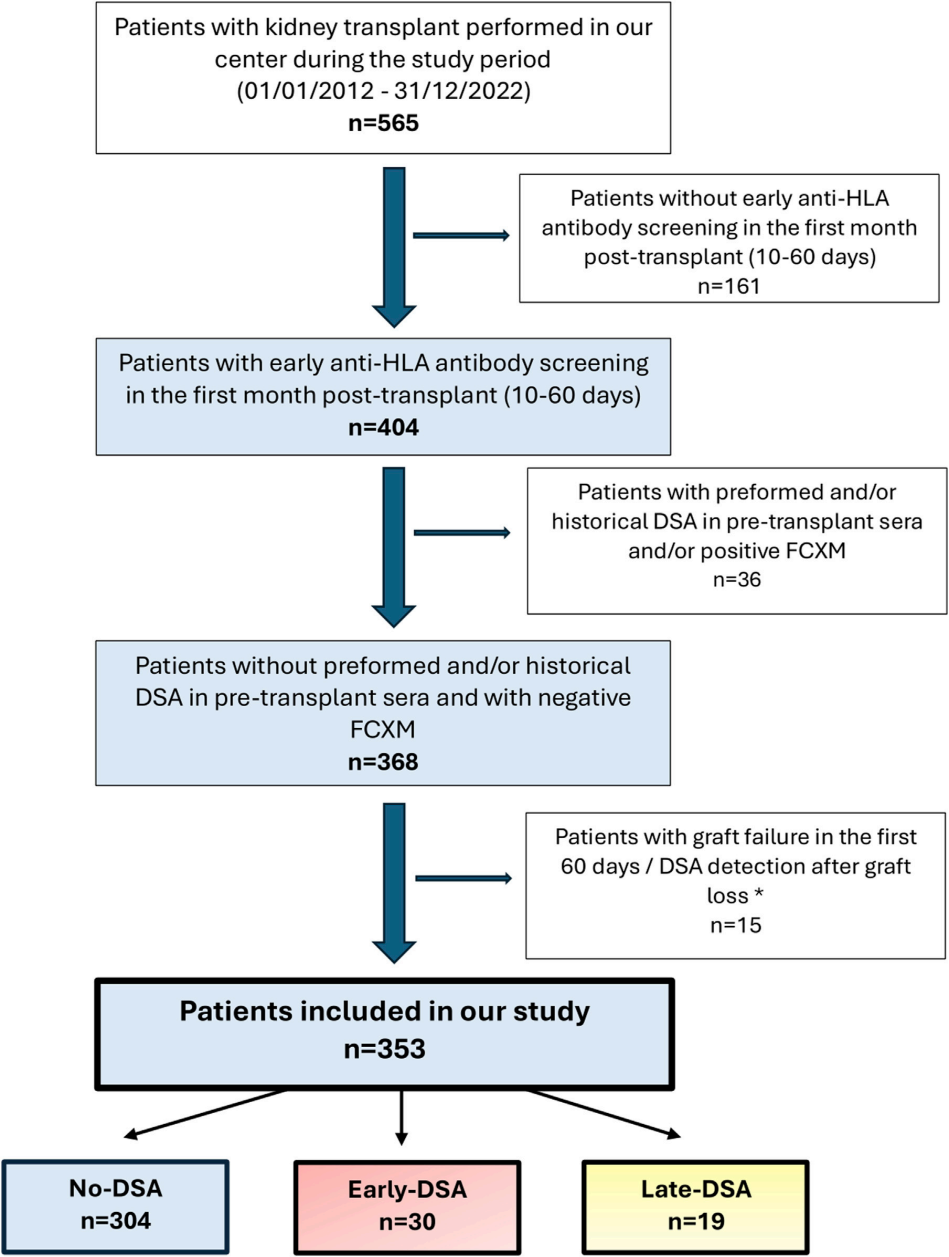
Flowchart of the patients included in our study. DSA: donor-specific antibodies. FCXM: flow cytometry crossmatch. *Causes of graft loss: graft bleeding requiring nephrectomy (6.7%, 1/15), death with functioning graft (13.3%, 2/15), hyperacute/acute rejection (20.0%, 3/15), arterial thrombosis (33.3%, 5/15) and venous thrombosis (26.7%, 4/15).

In total, we included 353 patients with early HLA antibody screening in the first month post-transplant (Figure 1). The time between transplant and last pre-transplant antibody sample was 42.0 days (IQR 16.0–73.0), and the median time from transplant to first anti-HLA antibody test was 30.0 days (IQR 26.0–37.0). Most patients (297, 84.1%) had systematic early HLA screening by protocol, and 56/353 (15.9%) underwent early HLA screening in the first month also for clinical indication (rise in creatinine and/or proteinuria). No significant differences were observed between DSA groups regarding early HLA antibody testing solely by protocol or by clinical indication (p = 0.178). At the time of early HLA antibody screening, the median creatinine (Cr) value was 1.49 mg/dL (IQR 1.13–1.97), the estimated glomerular filtration rate (eGFR) by CKD-EPI was 49.3 mL/min/1.73 m^2^ (IQR 34.4–67.1), and the median urine albumin-to-creatinine ratio was 69.6 mg/g (IQR 27.5–176.9). The time of initial hospital admission for transplant was 17.0 days (IQR 11.0–25.0), and most early HLA determinations in the first month (279/353, 79.0%) were performed on an outpatient basis, after first hospital admission.

The study cohort comprised mainly patients with a first single-kidney transplant from a deceased donor (Table 1) with a median follow-up of 3.8 years (IQR 2.1–6.4). By design of the study, none of the patients had pretransplant DSA and most patients were not sensitized (median cPRA 0.0%). Specifically analyzing patients with a first kidney transplant (n = 257, 72.8%), 20.6% had a pregnancy before transplantation. In this group of first transplants, pregnancy before transplant was significantly higher in sensitized patients with cPRA ≥5% (32.1% vs. 4.9%, p < 0.001) and cPRA ≥85% (7.5% vs. 0.5%, p = 0.001).

**TABLE 1:** Baseline characteristics of patients included in our study.

Baseline characteristics	All patients (n = 353)	No-DSA (n = 304)	Early-DSA (n = 30)	Late-DSA (n = 19)	p
Recipient age (years)^a^	57.0 (45.0–65.0)	58.0 (46.0–65.0)	49.5 (41.0–64.2)	45.0 (32.0–63.0)	0.235
Recipient sex (male) (n, %)^a^	245 (69.4%)	217 (71.4%)	17 (56.7%)	11 (57.9%)	0.133
Recipient sex (female) (n, %) • Pregnancy before transplant	108 (30.6%) 65 (60.2%)	87 (28.6%) 53 (60.9%)	13 (43.3%) 8 (61.5%)	8 (42.1%) 4 (50.0%)	0.133 0.829
Death-censored graft failure (n, %)	32 (9.1%)	19 (6.3%)	6 (20.0%)	7 (36.8%)	**<0.001**
Death (n, %)	37 (10.5%)	31 (10.2%)	5 (16.7%)	1 (5.3%)	0.406
Donor age (years)^a^	55.0 (45.0–63.0)	55.0 (45.0–63.0)	55.0 (44.7–63.2)	52.0 (41.0–69.0)	0.982
Donor sex (male) (n, %)	210 (59.5%)	180 (59.2%)	18 (60.0%)	12 (63.2%)	0.942
Donor type (deceased) (n, %) • DBD (n, %) • DCD (n, %)	337 (95.5%) 213 (60.3%) 124 (35.1%)	293 (96.4%) 183 (60.2%) 110 (36.2%)	27 (90.0%) 18 (60.0%) 9 (30.0%)	17 (89.5%) 12 (63.2%) 5 (26.4%)	0.120 0.542
Induction immunosuppression (n, %) • ATG (n, %) • Anti-IL2R (n, %)	253 (71.7%) 120 (47.4%) 133 (52.6%)	216 (71.1%) 92 (42.6%) 124 (57.4%)	23 (76.7%) 18 (78.3%) 5 (21.7%)	14 (73.7%) 10 (71.4%) 4 (28.6%)	0.793 **0.001**
First kidney transplant (n, %)	257 (72.8%)	228 (75.0%)	18 (60.0%)	11 (57.9%)	0.069
Retransplant (n, %) • Repeated HLA mismatch with previous donors (n, %)	96 (27.2%) 30 (31.3%)	76 (25.0%) 23 (30.3%)	12 (40.0%) 5 (41.7%)	8 (42.1%) 2 (25.0%)	0.069 0.675
Combined transplant (n, %) • Pancreas-kidney (n, %) • Liver-kidney (n, %)	23 (6.5%) 20 (87.0%) 3 (13.0%)	20 (6.6%) 17 (85.0%) 3 (15.0%)	2 (6.7%) 2 (100.0%) 0 (0.0%)	1 (5.3%) 1 (100.0%) 0 (0.0%)	0.974 0.772
CIT (hours)^a^	19.0 (10.0–23.0)	19.0 (10.0–23.0)	20.0 (11.2–22.2)	12.0 (5.0–22.0)	0.506
DGF (n, %)	91 (25.8%)	82 (27.0%)	6 (20.0%)	3 (15.8%)	0.746
Early blood transfusion (n, %)^b^	199 (56.4%)	169 (55.6%)	19 (63.3%)	11 (57.9%)	0.710
cPRA at the time of transplant (%)^a^	0.0 (0.0–0.0)	0.0 (0.0–0.0)	40.4 (0.0–87.8)	0.0 (0.0–36.3)	**<0.001**
cPRA ≥5% at the time of transplant (n, %)	79 (22.4%)	56 (18.4%)	17 (56.7%)	6 (31.6%)	**<0.001**
cPRA ≥85% at the time of transplant (n, %)	33 (9.3%)	24 (7.9%)	8 (26.7%)	1 (5.3%)	**0.003**
HLA-A mismatch >0 (n, %)	312 (88.4%)	266 (87.5%)	29 (96.7%)	17 (89.5%)	0.323
HLA-B mismatch >0 (n, %)	322 (91.2%)	277 (91.1%)	27 (90.0%)	18 (94.7%)	0.838
HLA-DRB1 mismatch >0 (n, %)	297 (84.1%)	257 (84.5%)	24 (80.0%)	16 (84.2%)	0.810
IS regimen at the time of early HLA screening (n, %) • Tacrolimus, MMF/MPA, prednisone • Tacrolimus, mTORi, prednisone • Others	344 (97.5%) 7 (2.0%) 2 (0.6%)	296 (97.4%) 7 (2.3%) 1 (0.3%)	29 (96.7%) 0 (0.0%) 1 (3.3%)	19 (100.0%) 0 (0.0%) 0 (0.0%)	0.748 0.562 0.106
Early HLA screening only by protocol (n, %)	297 (84.1%)	260 (85.5%)	22 (73.3%)	15 (78.9%)	0.178
Plasmapheresis before early HLA screening (n, %)^c^	35 (9.9%)	26 (8.6%)	7 (23.3%)	2 (10.5%)	**0.035**
IVIG before early HLA screening (n, %)^c^	30 (8.5%)	21 (6.9%)	7 (23.3%)	2 (10.5%)	**0.008**
ABMR (Banff 2019 Classification) (n, %)^d^ • Time from transplant to ABMR (months)^a^	69 (19.5%) 3.3 (0.6–12.1)	38 (12.5%) 2.7 (0.5–12.0)	16 (53.3%) 1.7 (0.5–3.6)	15 (78.9%) 11.4 (1.5–35.7)	**<0.001** **0.027**
Biopsy with ABMR diagnosis by clincal indication or 1-year protocol • Clinical indication (graft dysfunction and/or DSA appearance) (n, %) - Only for DSA (n, %) - Graft dysfunction (n, %) • 1-year-Protocol (n, %)	54 (78.3%) 15 (21.7%)	26 (68.4%) 12 (31.6%)	16 (100.0%) 5 (31.3%) 11 (68.8%) 0 (0.0%)	12 (80.0%) 2 (16.7%) 10 (83.3%) 3 (20.0%)	**0.036**
TCMR (Banff 2019 Classification) (n, %)^d^	96 (27.2%)	75 (24.7%)	11 (36.7%)	10 (52.6%)	**0.014**

^a^ Median and interquartile range. For continuous variables with non-normal distribution, the Kruskal-Wallis test was used to compare the 3 groups.

^b^ Early blood transfusion: at least one blood transfusion within the first 30 days after transplant.

^c^ Treatment with plasmapheresis and/or IVIG, before the first early HLA, determination was performed as rejection prophylaxis in high-risk patients, suspected rejection and inability to perform a biopsy, or biopsy-proven ABMR.

^d^ Rejection episodes were categorized according to Banff 2019 Classification. Borderline rejection was included in the category of T-cell mediated rejection (TCMR).

DBD: donor brain death. DCD: donor circulatory death. ATG, antithymocyte globulin; Anti-IL2R, anti-interleukin-2, receptor. CIT: cold ischemia time. DGF: delayed graft-function. cPRA: calculated panel-reactive antibody (Eurotransplant). IS: immunosuppression. MMF/MPA: Mycophenolate mofetil/Mycophenolic acid. mTORi: mTOR, inhibitor. IVIG: Intravenous immunoglobulin. ABMR: antibody-mediated rejection. TCMR: T-cell mediated rejection.

Graft failure occurred in 9.1% of patients and 19.5% had ABMR. Delta cPRA >0% developed in 54 patients (15.3%) whereas 30 out of 353 (8.5%) had early-DSA detection in the first month, and the total number of patients with DSA appearance during the complete follow-up period was 49/353 (13.9%). In our cohort, the median time from transplant to first DSA detection was 1.5 months (IQR 1.0–11.4) (Supplementary Figure S1).

When evaluating the characteristics of patient groups (Table 1), they were comparable in terms of recipient age, recipient sex, donor age and donor type. 71.7% of patients received induction immunosuppression, with more induction with anti-thymocyte globulin (ATG) in the early-DSA group (p = 0.001). Patients in the early-DSA group had more HLA sensitization at the time of transplant compared to patients with late-DSA and no-DSA (cPRA 40.4% vs. 0.0% vs. 0.0%, p < 0.001). There was a higher proportion of patients receiving plasmapheresis and intravenous immunoglobulin (IVIG) treatment before early screening in the early-DSA group (p = 0.035 and p = 0.008, respectively). There were differences between groups in T-cell mediated rejection (TCMR) episodes, with a higher incidence in patients with late-DSA (p = 0.014). Patients with early-DSA and late-DSA had more proportion of ABMR compared to patients without DSA (53.3% vs. 78.9% vs. 12.5%, p < 0.001), and the median time from transplant to first ABMR episode was shorter in the early-DSA group (p = 0.027). A higher proportion of biopsies with ABMR diagnosis were performed for clinical indication in the early and late-DSA groups (p = 0.036). Histological data are shown in Supplementary Tables S1–S3.

### Patients With Early-DSA

Most patients with early-DSA had class-I (56.7%), 33.3% had class-II and 10.0% had class-I and class-II. Regardless of the antibody class, 33.3% had >1 DSA in the same first serum after transplant. The median MFI level at first early-DSA detection was 4,912.0 (IQR 2,505.7–7,235.2). “Transient” early-DSA were detected in 20/30 patients (66.7%), and 60.0% (12/20) had early-DSA negativity after rejection and specific active treatment whereas 40.0% (8/20) had “spontaneous” disappearance of early-DSA without treatment for rejection.

The median values of estimated glomerular filtration rate and urine albumin-creatinine ratio in the early-DSA group at the time of early-DSA appearance were 37.5 mL/min/1.73 m^2^ (IQR 28.7–60.0) and 91.0 mg/g (IQR 43.8–332.7), respectively. At least one allograft biopsy was performed in 83.3% of patients with early-DSA. Technical difficulty, high-risk due to anticoagulation, patient refusal or “transient” early-DSA without evidence of graft dysfunction were the reasons for not performing a biopsy in 5/30 patients. ABMR was present in 53.3% of patients with early-DSA, and 46.7% had “subclinical” early-DSA without evidence of ABMR. In patients with ABMR, 31.3% (5/16) had rejection before early-DSA appearance in the first month and 68.8% (11/16) presented ABMR at the time or after early-DSA detection. Patients with early-DSA and ABMR were associated with lower allograft survival compared to those patients with “subclinical” early-DSA (log rank p = 0.012) (Supplementary Figure S2). 36.7% of patients with early-DSA (11/30) presented TCMR, and 36.4% of these patients (4/11) had TCMR before first early-DSA appearance. The characteristics of DSA in patients with early-DSA and late-DSA are illustrated in Table 2. The evolution of eGFR in patients with DSA is shown in Supplementary Table S4 and Supplementary Figures S3, S4.

**TABLE 2:** DSA characteristics in patients with early-DSA and late-DSA.

DSA characteristics	Early-DSA (n = 30)	Late-DSA (n = 19)	p
Class of DSA (n, %) • Class-I • Class-II^a^ • Both class-I and class-II^a^	17 (56.7%) 10 (33.3%) 3 (10.0%)	6 (31.6%) 10 (52.6%) 3 (15.8%)	0.230
Number of DSA per patient: >1 DSA in the first sample (n, %)	10 (33.3%)	7 (36.8%)	0.801
“Transient” DSA (n, %)	20 (66.7%)	7 (36.8%)	**0.041**
MFI at DSA first occurrence^b^	4912.0 (2505.7–7235.2)	2428.5 (1432.5–11,109.0)	0.365
IS at the time of first DSA detection • Tacrolimus (n, %) • MMF/MPA (n, %) • Prednisone (n, %) • mTORi (n, %)	29 (96.7%) 29 (96.7%) 29 (96.7%) 0 (0.0%)	18 (94.7%) 18 (94.7%) 17 (89.5%) 2 (10.5%)	0.739 0.739 0.306 0.070
ABMR (Banff 2019 Classification) (n, %) • ABMR before DSA detection • ABMR at the time/after DSA detection Time from DSA to ABMR diagnosis (months)^b^	16 (53.3%) 5 (31.3%) 11 (68.8%) 1.4 (0.3–3.1)	15 (78.9%) 7 (46.7%) 8 (53.3%) 3.2 (2.0–5.2)	0.070 0.370

^a^ Of patients with class-II DSA, in both groups (alone or together with class-I DSA), 18/26 (69.2%) had anti-DQB, 5/26 (19.2%) had anti-DQA, and 1/16 (3.8%) had anti-DP DSA.

^b^ Median and interquartile range. For continuous variables with non-normal distribution, the Mann-Whitney U test was used to compare the 2 groups.

DSA: donor-specific HLA, antibody. MFI: mean fluorescence intensity. IS: immunosuppression. MMF/MPA: Mycophenolate mofetil/Mycophenolic acid. mTORi: mTOR, inhibitor.

### DSA Status, Rejection and Graft Survival

**FIGURE 2:**
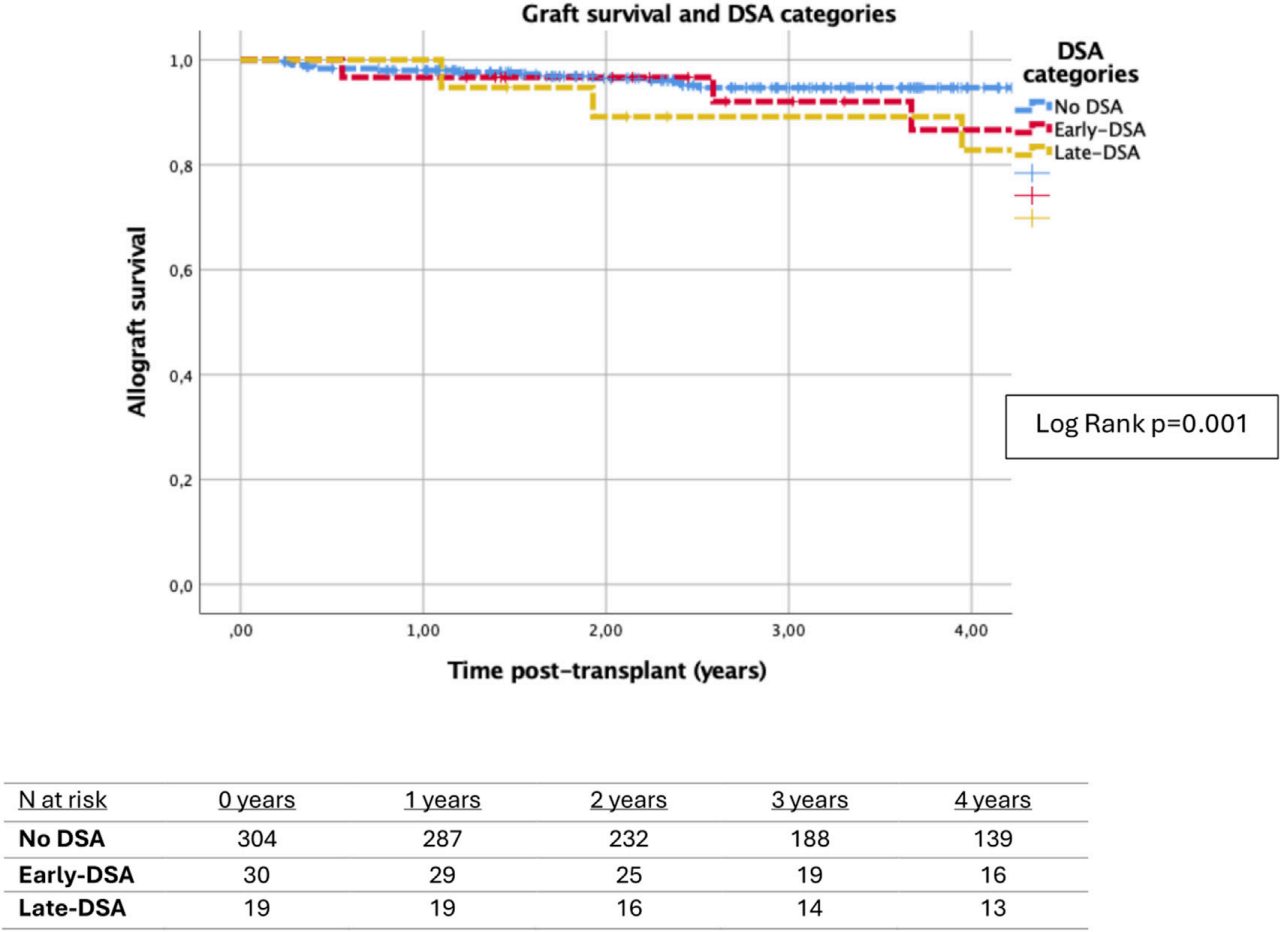
Kaplan-Meier survival analysis of death-censored graft failure for DSA categories. Patients with early-DSA and late-DSA were associated with lower allograft survival compared to patients without DSA (log rank p = 0.001). Graft survival at 3 years posttransplant was 94.7% (± 1.4%) in no-DSA patients, 92.1% (5.5%) in patients with early-DSA in the first month and 89.2% (± 7.2%) in patients with late-DSA.

The presence of early-DSA and late-DSA was associated with lower death-censored allograft survival (log rank p = 0.001), as shown in Figure 2. Independently of DSA status, patients with ABMR were associated with lower allograft survival compared to patients without ABMR (log rank p = 0.001). Similarly, the presence of TCMR was associated with lower graft survival (log rank p = 0.006). These results are shown in Supplementary Figures S5, S6.

**FIGURE 3:**
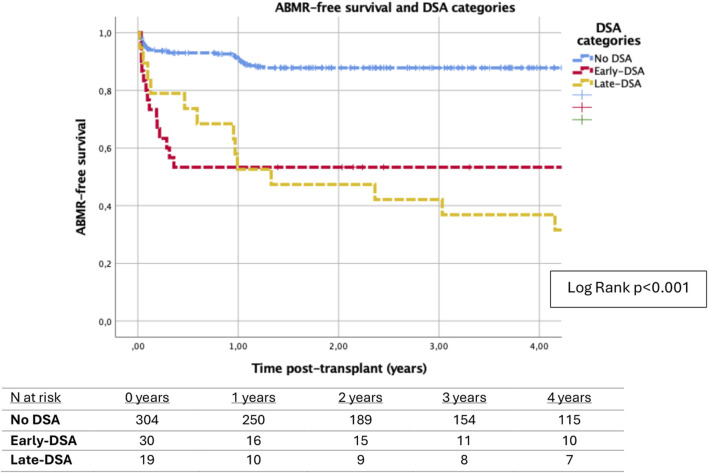
Kaplan-Meier survival analysis of antibody-mediated rejection (ABMR) for DSA categories. Patients with early-DSA and late-DSA were associated with lower ABMR-free survival compared to patients without DSA (log rank p < 0.001). At 3 years posttransplant, ABMR-free survival was 87.8% (± 1.9%) in no-DSA patients, 53.3% (9.1%) in patients with early-DSA, and 42.1% (11.3%) in patients with late-DSA.

Analyzing the relationship between DSA and ABMR, the presence of DSA was associated with lower ABMR-free survival (log rank p < 0.001) (Figure 3). Different patient characteristics were associated with ABMR in univariable Cox regression analyses (Table 3). Specifically analyzing factors that were associated with ABMR posttransplant by multivariable Cox regression (Table 4), early-DSA and late-DSA were independent risk factors for ABMR (HR 3.3, CI 95% 1.6–6.8, p = 0.001 and HR 4.1, CI 95% 1.7–9.5, p = 0.001, respectively). Conversely, first kidney transplant, DGF or HLA sensitization at the time of transplant were not contributors in the multivariable model.

**TABLE 3:** Univariable Cox regression analysis for antibody-mediated rejection (ABMR).

*Univariable cox regression for ABMR* ^a^	HR	CI 95% INF	CI 95% SUP	p
Recipient age	0.985	0.966	1.006	0.158
Donor age	0.982	0.964	1.001	0.061
Deceased donor	2.000	0.798	5.015	0.139
DCD	0.910	0.519	1.519	0.743
Induction immunosuppression	1.478	0.795	2.747	0.216
First kidney transplant	0.523	0.308	0.888	**0.016**
Combined transplant	0.645	0.201	2.068	0.461
CIT	1.022	0.986	1.059	0.235
DGF	1.779	1.026	3.083	**0.040**
cPRA at the time of transplant	1.009	1.003	1.016	**0.007**
HLA-A mismatch >0	1.506	0.601	3.773	0.382
HLA-B mismatch >0	1.237	0.448	3.418	0.682
HLA-DRB1 mismatch >0	2.059	0.822	5.157	0.123
HLA mismatch – sum of A, B and DRB1 mismatch	1.069	0.884	1.293	0.490
Early-DSA	3.279	1.696	6.339	**<0.001**
Late-DSA	3.981	1.882	8.421	**<0.001**

ABMR: antibody-mediated rejection. DCD: donor circulatory death. CIT: cold ischemia time. DGF: delayed graft-function. cPRA: calculated panel-reactive antibody (Eurotransplant). DSA: donor-specific HLA, antibody.

^a^ To evaluate the predictive capacity of DSA, by Cox regression, patients with ABMR before DSA appearance were eliminated from this analysis.

**TABLE 4:** Multivariable Cox regression analysis for antibody-mediated rejection (ABMR).

*Multivariable cox regression for ABMR* ^a^	HR	CI 95% INF	CI 95% SUP	p
First kidney transplant	0.854	0.423	1.722	0.659
DGF	1.696	0.963	2.986	0.067
cPRA at the time of transplant	1.005	0.996	1.014	0.308
Early-DSA	3.376	1.661	6.864	**0.001**
Late-DSA	4.122	1.785	9.518	**0.001**

ABMR: antibody-mediated rejection. DGF: delayed graft-function. cPRA: calculated panel-reactive antibody (Eurotransplant). DSA: donor-specific HLA, antibody.

^a^ To evaluate the predictive capacity of DSA, by Cox regression, patients with ABMR before DSA appearance were eliminated from this analysis.

## Discussion

Despite the widely described impact of DSA on graft outcomes in kidney transplantation [1–6], the indication of universal post-transplant HLA antibody screening remains unclear [26, 29]. Data on the cost-effectiveness of DSA monitoring are scarce, and different strategies have been proposed to select high-risk patients [27, 28]. The frequency of HLA screening is also not determined and current recommendations have a low level of evidence [15, 29, 30]. In our center we established regular DSA screening in the first month posttransplant more than 10 years ago and in this study, we describe a large cohort of mostly non-sensitized patients without preformed or historical DSA and with early HLA antibody screening by Luminex in the first month. After 3.8 years of follow-up, DSA develop in 13.9% of patients with a median time to first DSA appearance of 1.5 months post-transplantation. Early-DSA are predominant (8.5%) and are an independent risk factor for ABMR. Patients with early-DSA had more HLA sensitization at the time of transplant, presumably reflecting alloimmune memory even in the absence of preformed DSA, therefore we suggest that early-DSA screening should be performed in this high-risk population. Consequently, with our data we highlight that assessing the HLA sensitization status and immunological risk of patients may be the best tool to generate a post-transplant DSA monitoring scheme.

The timing of DSA appearance after transplantation is variable and most series have described a higher incidence in the first year, ranging from 3% to 20%, with a lower annual rate thereafter [17, 29, 33]. A recent study showed that 77% of DSA detected by screening appeared in the first 100 days post-transplant [32]. However, some series have shown that dnDSA can appear up to 10 years after transplant [16, 29], demonstrating that the risk of developing DSA should be considered at any time during functional graft life. In our cohort, the incidence of DSA is almost 14% (49/353) over about 4 years of follow-up, and although the number of patients with DSA is not very high, our incidence is in line with previous observations [5, 34, 42]. Surprisingly, most of them (30/49) are detected early in the first month. These results are probably explained by the more intensive monitoring in our center with a first HLA determination in the first month, allowing rapid detection of DSA. A relevant proportion of patients with early-DSA (8/30) present “spontaneous” negativity at 3 or 6 months after first appearance, and these “transient” early-DSA cannot be detected if first early HLA determination is omitted, which could explain our high incidence. Also, our study describes two exclusive categories of patients based on first detection of DSA, and patients of the early-DSA group may develop new DSA later after transplantation. Despite these considerations, our study shows a relevant proportion of patients with DSA appearance in the first month, emphasizing that early HLA screening allows prompt detection of patients at potential risk of poor outcomes.

The STAR Working Group noted that DSA up to the third month after transplant are likely preformed and reflect alloimmune memory [15, 25]. Previous transplants are a risk factor for memory responses [15] and in patients undergoing retransplantation it has been described that re-exposure to mismatched HLA antigens may be associated with increased immunological risk [43]. In our cohort there are no differences between groups in the proportion of first transplants. 31.3% of our retransplanted patients present a repeated HLA mismatch with previous donors, without differences between patient groups, being in line with previous observations showing that a repeated HLA mismatch within negative FCXM and without described preformed DSA is not associated with DSA detection after transplant [44]. The presence of non-DSA HLA antibodies at the time or before transplantation may also be a risk factor for latent alloimmune memory responses [15]. In our study, patients with HLA sensitization present a higher risk of early-DSA detection in the first month, probably reflecting a memory response even with a negative FCXM and in the absence of preformed DSA. ATG induction is higher in the early-DSA group, possibly explained by the greater number of highly sensitized patients. Because of this high risk, patients with early-DSA also more frequently received plasmapheresis and/or IVIG prior to early HLA screening in the first month and early-DSA detection, showing that current treatments are not fully effective in suppressing DSA. Despite this, there is an important proportion of non-sensitized patients (cPRA = 0% in 12/30) who develop DSA rapidly in the first month. These data likely show that the percentage of cPRA in an isolated serum is not completely informative about the HLA sensitization status or the risk of memory responses, and a cPRA of 0% does not necessarily represent an immunologically naïve patient [15]. Patients with prior exposure to alloantigens through previous transplants, pregnancies or transfusions may not have detectable anti-HLA antibodies, and preexisting DSA below the positivity threshold could be present but not detected by initial screening [13] and triggered by transplantation. In fact, to study alloimmune memory beyond the presence or absence of anti-HLA antibodies, other immune assays that detect donor-specific B and T cell memory, such as the interferon-γ enzyme-linked immunospot (ELISpot), have been described as a monitoring tool to assess cellular immune risk [45–48]. Therefore, with our data we highlight the complexity of the clinical measurement of immune memory and the importance of stratifying the immunological risk of patients to predict the risk of developing DSA.

The development of ABMR regardless of DSA status is significantly associated with lower graft survival in our cohort (log rank p = 0.001), emphasizing ABMR as a fundamental cause of allograft failure, as widely described [1–6]. In our study, more than half of the patients with ABMR (55.0%) did not have detectable DSA, highlighting that the presence of C4d staining in the biopsy is a sufficient factor that allows ABMR diagnosis without serological evidence of DSA [36, 49]. The presence of DSA is associated with ABMR and graft failure, however the clinical evolution of patients with DSA is variable [50]. The MFI level may have predictive capacity, but the relationship of a single MFI value with clinical outcomes is not established, and some characteristics of DSA such as class, specificity, IgG subclass or complement binding capacity may be prognostic factors [16, 17, 51–53]. In our cohort, 19.5% of patients develop ABMR, with a higher incidence in patients with DSA. Despite this, almost half of patients with early-DSA have “subclinical” DSA without evidence of rejection and 26.6% present “transient” early-DSA with “spontaneous” disappearance, underscoring the different clinical course of patients with DSA and the urgent need to improve our knowledge of DSA characteristics and prognostic factors to determine patients at highest risk of rejection after DSA detection [50, 53].

Our study shows early-DSA as a determining factor of poor outcomes, since the presence of early-DSA is associated with a 3.3-fold higher risk of ABMR in multivariable analysis independently of other variables such as DGF, first kidney transplant or HLA sensitization. Furthermore, the presence of late-DSA is significantly associated with lower allograft survival (log rank p = 0.001) and ABMR-free survival (log rank p < 0.001), and remains a strong, independent risk factor for ABMR in multivariable analysis (HR 4.1). Altogether, our data support that the presence of DSA at any time after transplant is a consistent risk factor for ABMR, with worse outcomes in the group of patients with late-DSA. It has been described that class-II DSA usually appear later after transplant and are the most common dnDSA, being more harmful and resistant to treatment [16, 54]. Our group of patients with late-DSA presents a higher percentage of class-II, the proportion of “transient” DSA is lower as well as the number of “subclinical” DSA, and they have more chronic ABMR changes, showing a different profile of patients with DSA that could explain our findings and determine the worse outcomes compared to the early-DSA group. It has been shown that patients with dnDSA present lower allograft survival compared to preexisting DSA [19]. Moreover, it has been demonstrated that patients with preformed DSA that persist after transplant have a higher incidence of ABMR than patients without DSA, but with a significantly lower risk of ABMR compared to those with dnDSA [55]. Accordingly, these results support our hypothesis that early-DSA could represent a memory response and have a better outcome than late-DSA.

The indication and frequency of universal HLA screening are not established, and it has been proposed a monitoring scheme with a first determination at 3–6 months post-transplant [29]. Although it seems that monitoring of preformed DSA may be useful [29, 56], high-quality data are lacking. It has been described that an early determination in the first month may be necessary in intermediate-risk patients with historical DSA or HLA sensitization [30], but there are currently no clear guidelines and the STAR 2017 Working Group suggests an early post-transplant HLA assessment in patients at risk for latent memory responses, without making specific recommendations on frequency and duration due to the absence of robust evidence [15]. In our cohort, early-DSA are consistently associated with ABMR and appear more frequently in sensitized patients. Most patients with early-DSA (68.8%) present ABMR at the time or after DSA detection. The time to ABMR diagnosis is shorter in patients with early-DSA compared to those with late-DSA (1.7 vs. 11.4 months), demonstrating that early screening potentially identifies high-risk patients and reduces the time to ABMR diagnosis, allowing prompt therapy initiation. Hence, until the clinical measurement of immune memory is better known and implemented in clinical routine to assess immunological risk, we suggest that early-DSA monitoring in the first month should be performed at least in patients with HLA sensitization, without waiting for the third or sixth month.

It should be noted that there is a time between the initial follow-up in our cohort and the determination of anti-HLA antibodies in which the appearance of DSA is not possible, mainly for analytical reasons, since there is a period without measurement of anti-HLA antibodies. Of note, all patients included in our study had a recent anti-HLA antibody sample prior to transplantation (median 42 days), and all patients underwent anti-HLA antibody testing at a specific time point or “landmark,” the first month (median 30 days). Therefore, we closely monitor pre- and post-transplant HLA antibodies, and we perform a very early first HLA determination after transplant, which allows us to correctly assess the clinical impact of DSA on graft outcomes.

Nevertheless, our study has several limitations. This is a single-center, retrospective analysis that includes patients with HLA monitoring by Luminex, with a limited sample size and a low number of patients with DSA during the follow-up period. Our study presents a cohort of patients without preformed DSA and with negative FCXM, but with a variable risk of memory responses due to the inclusion of women with previous pregnancies, retransplants, and different degrees of HLA sensitization; despite this, the fact that our cohort is heterogeneous emphasizes our fundamental finding that early-DSA appearance represents memory and allows us to identify patients at risk. Although it is the best technique available, Luminex has multiple limitations and in our center SAB assay is only performed if initial screening is positive. The MFI positivity threshold is set at 1,500 as widely accepted in the literature, however the MFI cut-off value is controversial and can be affected by several parameters. The MFI level at the time of DSA detection is recorded but not the evolution of MFI in subsequent samples. Some characteristics of DSA that may be related to poor outcomes, such as complement binding capacity, are not analyzed as they are not performed routinely. Adherence to treatment and levels of immunosuppressive drugs are also not evaluated at the time of DSA detection. Pretransplant history of transfusions to assess immune memory is not registered. Classical antigen HLA mismatch is considered, without analyzing epitope mismatch. The lack of complete donor typing, especially in DP and DQ, does not allow us to rule out previous sensitization and limits the definition of post-transplant DSA at these HLA loci. To calculate repeated HLA mismatch in retransplants, it must be noted that most previous donor typings are low resolution, and repeated mismatches are not assessed at a molecular level. Finally, the presence of non-HLA antibodies is not analyzed. The fundamental strength of our study is having a large and well-described cohort of patients with early HLA determination in the first month posttransplant. Furthermore, we have clinical and histological data available that allow us to evaluate the impact of early-DSA on graft outcomes and the clinical course of patients after routine early HLA monitoring.

While more accurate knowledge of immunological risk and clinical measurement of immune memory is needed, our study describes a relevant proportion of patients with DSA detection in the first month (8.5%), probably showing alloimmune memory even in the absence of preformed DSA described in pretransplant sera and in the context of negative FCXM. Although almost half of these early-DSA are ‘subclinical’ without evidence of humoral injury, the presence of early-DSA remains a consistent risk factor for ABMR. The risk of developing early-DSA is significantly higher in patients with HLA sensitization, therefore routine early HLA screening in the first month may be reasonably performed in these high-risk patients. In conclusion, we provide granular evidence on the impact of early-DSA detected by screening on clinical events, being strongly related to ABMR and inferior outcomes. High-quality data on the clinical course of patients with DSA detected by universal screening and cost-effectiveness studies are essential to improve results and provide an appropriate post-transplant DSA monitoring strategy.

## Data Availability

The original contributions presented in the study are included in the article/Supplementary Material, further inquiries can be directed to the corresponding author.
