# Supplementary material for: Early Donor-Specific HLA Antibodies Detected by Screening in the First Month Posttransplant and Kidney Graft Outcomes
Source: Transpl Int. 2025 Aug 6;38:14424. doi: 10.3389/ti.2025.14424 (PMC12364724; doi:10.3389/ti.2025.14424)
Supplement: Supplementary file 1 [file DataSheet1.docx]

**Supplementary Material**

**Supplementary Figure 1. Time from transplant to first DSA appearance.**

In patients with DSA in our cohort (n=49), the median time from transplant to first DSA detection is 1.5 months (IQR 1.0-11.4).

**
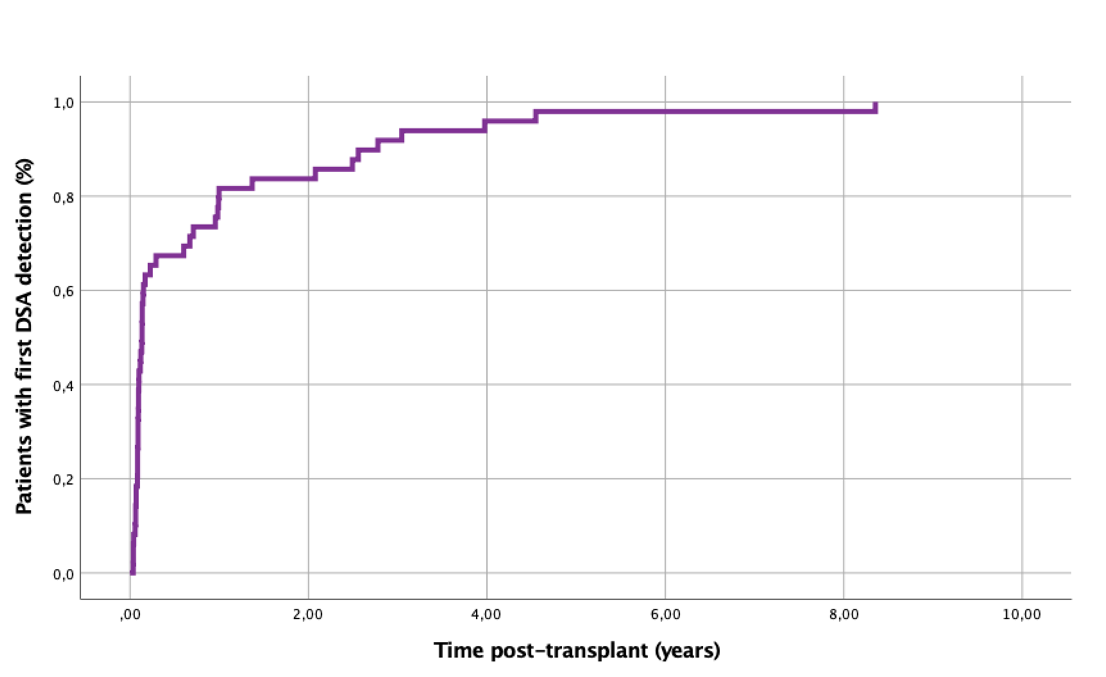
**

**Supplementary Table 1. Patients with ABMR and Banff categories.**

**^** Rejection episodes were categorized according to Banff 2019 Classification.

|  | **All patients**  **(n=353)** | **No-DSA**  **(n=304)** | **Early-DSA**  **(n=30)** | **Late-DSA**  **(n=19)** | **p** |
| --- | --- | --- | --- | --- | --- |
| **ABMR (Banff 2019 Classification) (n, %)^**  **ABMR categories:**  **- Active ABMR (n, %)**  **- Chronic active ABMR (n,%)**  **- Chronic ABMR (n, %)** | 69 (19.5%)  56 (81.2%)  13 (18.8%)  0 (0.0%) | 38 (12.5%)  31 (81.6%)  7 (18.4%)  0 (0.0%) | 16 (53.3%)  16 (100.0%)  0 (0.0%)  0 (0.0%) | 15 (78.9%)  9 (60.0%)  6 (40.0%)  0 (0.0%) | **<0.001**  **0.017** |
| **ABMR acute Banff score ‘g’**  **- 0**  **- 1**  **- 2**  **- 3** | - 11 (15.9%)  - 38 (55.1%)  17 (24.6%)  - 3 (4.3%) | - 6 (15.8%)  - 23 (60.5%)  - 9 (23.7%)  - 0 (0.0%) | - 3 (18.8%)  - 8 (50.0%)  - 4 (25.0%)  - 1 (6.3%) | - 2 (13.3%)  - 7 (46.7%)  - 4 (26.7%)  - 2 (13.3%) | 0.515 |
| **ABMR acute Banff score ‘ptc’**  **- 0**  **- 1**  **- 2**  **- 3** | - 15 (21.7%)  - 30 (43.5%)  - 19 (27.5%)  - 5 (7.2%) | - 9 (23.7%)  - 19 (50.0%)  - 9 (23.7%)  - 1 (2.6%) | - 3 (18.8%)  - 5 (31.3%)  - 6 (37.5%)  - 2 (12.5%) | - 3 (20.0%)  - 6 (40.0%)  - 4 (26.7%)  - 2 (13.3%) | 0.623 |
| **ABMR acute Banff score ‘v’**  **- 0**  **- 1**  **- 2**  **- 3** | - 55 (79.7%)  - 13 (18.8%)  - 1 (1.4%)  - 0 (0.0%) | - 31 (81.6%)  - 7 (18.4%)  - 0 (0.0%)  - 0 (0.0%) | - 14 (87.5%)  - 2 (12.5%)  - 0 (0.0%)  - 0 (0.0%) | - 10 (66.7%)  - 4 (26.7%)  - 1 (6.7%)  - 0 (0.0%) | 0.299 |
| **ABMR acute Banff score ‘C4d’**  **- 0**  **- 1**  **- 2**  **- 3** | - 3 (4.3%)  - 38 (55.1%)  - 18 (26.1%)  - 10 (14.5%) | - 0 (0.0%)  - 25 (65.8%)  - 12 (31.6%)  - 1 (2.6%) | - 2 (12.5%)  - 6 (37.5%)  - 4 (25.0%)  - 4 (25.0%) | - 1 (6.7%)  - 7 (46.7%)  - 2 (13.3%)  - 5 (33.3%) | **0.013** |

**Supplementary Table 2. Patients with TCMR and Banff categories.**

**^** Rejection episodes were categorized according to Banff 2019 Classification. Borderline rejection was included in the category of T-cell mediated rejection (TCMR).

|  | **All patients**  **(n=353)** | **No-DSA**  **(n=304)** | **Early-DSA**  **(n=30)** | **Late-DSA**  **(n=19)** | **p** |
| --- | --- | --- | --- | --- | --- |
| **TCMR (Banff 2019 Classification) (n, %)^**  **TCMR categories (n,%):**  **- Acute TCMR borderline**  **- Acute TCMR grade IA**  **- Acute TCMR grade IB**  **- Acute TCMR grade IIA**  **- Acute TCMR grade IIB**  **- Acute TCMR III** | 96 (27.2%)  48 (50.0%)  11 (11.5%)  10 (10.4%)  23 (24.0%)  2 (2.1%)  2 (2.1%) | 75 (24.7%)  36 (48.0%)  9 (12.0%)  9 (12.0%)  18 (24.0%)  1 (1.3%)  2 (2.7%) | 11 (36.7%)  10 (90.9%)  0 (0.0%)  0 (0.0%)  1 (9.1%)  0 (0.0%)  0 (0.0%) | 10 (52.6%)  2 (20.0%)  2 (20.0%)  1 (10.0%)  4 (40.0%)  1 (10.0%)  0 (0.0%) | **0.014**  **0.004**  0.339  0.477  0.253  0.173  0.751 |
| **TCMR acute Banff score ‘i’ (n, %)**  **- 0**  **- 1**  **- 2**  **- 3** | - 4 (4.2%)  - 53 (55.2%)  - 30 (31.3%)  - 9 (9.4%) | - 3 (4.0%)  - 42 (56.0%)  - 23 (30.7%)  - 7 (9.3%) | - 1 (9.1%)  - 8 (72.7%)  - 1 (9.1%)  - 1 (9.1%) | - 0 (0.0%)  - 3 (30.0%)  - 6 (60.0%)  - 1 (10.0%) | 0.301 |
| **TCMR acute Banff score ‘t’ (n, %)**  **- 0**  **- 1**  **- 2**  **- 3** | - 1 (1.0%)  - 48 (50.0%)  - 29 (30.2%)  - 18 (18.8%) | - 1 (1.3%)  - 40 (53.3%)  - 20 (26.7%)  - 14 (18.7%) | - 0 (0.0%)  - 4 (36.4%)  - 7 (63.6%)  - 0 (0.0%) | - 0 (0.0%)  - 4 (40.0%)  - 2 (20.0%)  - 4 (40.0%) | 0.114 |
| **TCMR acute Banff score ‘v’ (n, %)**  **- 0**  **- 1**  **- 2**  **- 3** | - 69 (71.9%)  - 23 (24.0%)  - 2 (2.1%)  - 2 (2.1%) | - 54 (72.0%)  - 18 (24.0%)  - 1 (1.3%)  - 2 (2.7%) | - 10 (90.9%)  - 1 (9.1%)  - 0 (0.0%)  - 0 (0.0%) | - 5 (50.0%)  - 4 (40.0%)  - 1 (10.0%)  - 0 (0.0%) | 0.293 |

**Supplementary Table 3. Patients with DSA and without ABMR. Banff scores and microvascular inflammation (MVI).**

**^** Rejection episodes were categorized according to Banff 2019 Classification.

* In cases of moderate MVI (g + ptc ≥ 2), the criterion of ‘ptc ≥ 2 alone is not sufficient and g must be ≥1’ of the Banff 2019 Classification was not met.

|  | Early-DSA (n=30) | Late-DSA  (n=19) | p |
| --- | --- | --- | --- |
| No ABMR (n, %) ^  No ABMR, with biopsy (n, %)  ‘g’:  - 0 (n, %)  - 1 (n, %)  - 2 (n, %)  - 3 (n, %)  ‘ptc’:  - 0 (n, %)  - 1 (n, %)  - 2 (n, %)  - 3 (n, %)  ‘g + ptc’ ≥ 2 (n, %) * | 14 (46.7%)  9 (64.3%)  - 7 (77.8%)  - 0 (0.0%)  - 2 (22.2%)  - 0 (0.0%)  - 6 (66.7%)  - 1 (11.1%)  - 2 (22.2%)  - 0 (0.0%)  3 (33.3%) | 4 (21.1%)  4 (100.0%)  - 2 (50.0%)  - 2 (50.0%)  - 0 (0.0%)  - 0 (0.0%)  - 4 (100.0%)  - 0 (0.0%)  - 0 (0.0%)  - 0 (0.0%)  0 (0.0%) | 0.160  0.058  0.420  0.188 |

**Supplementary Figure 2. Kaplan-Meier survival analysis for death-censored graft failure of early-DSA.** Patients with early-DSA and antibody-mediated rejection (ABMR) were associated with lower allograft survival compared to those patients with ‘subclinical’ early-DSA (log rank p=0.012).


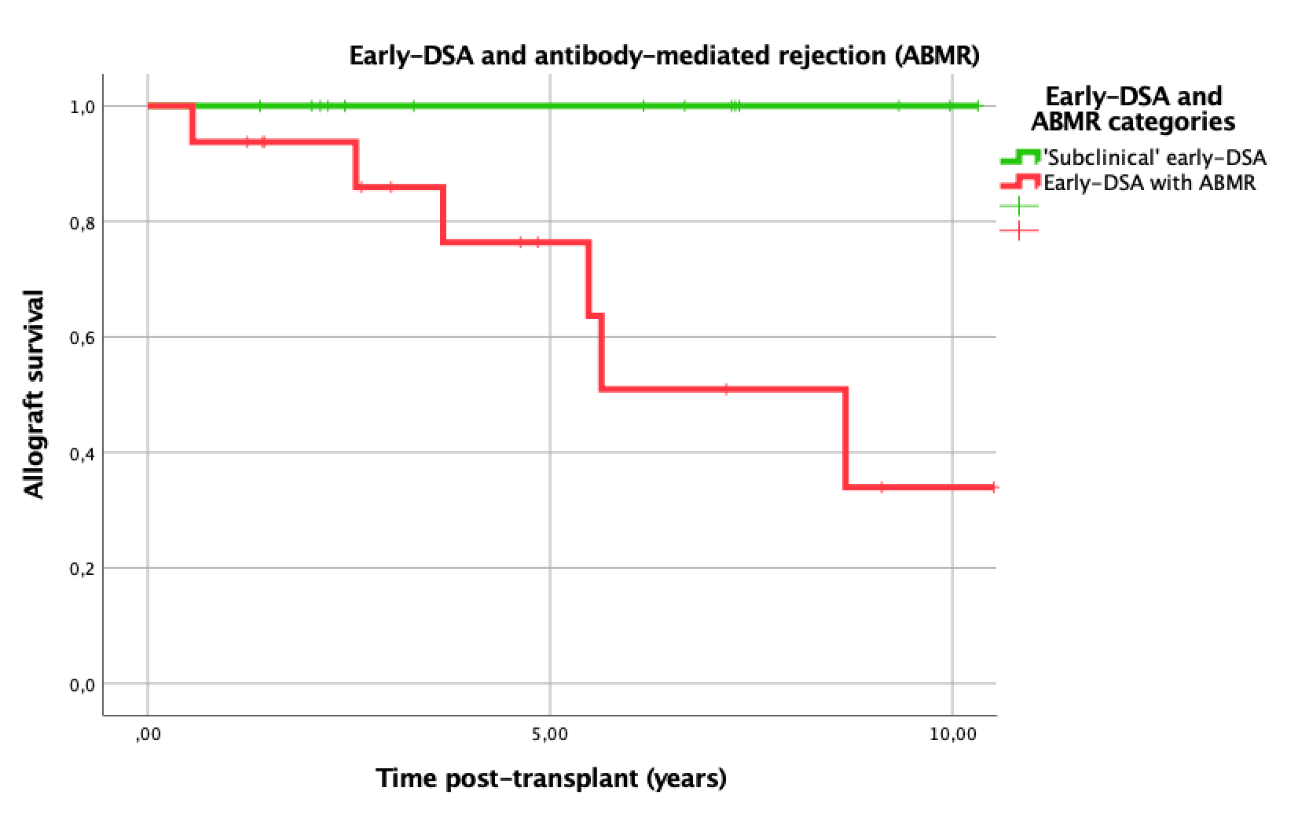


Log Rank p=0.012

**Supplementary Table 4. Patients with DSA and estimated glomerular filtration rate (eGFR) values during follow-up.**

* Mean and standard deviation (SD). T-Student test to compare variables with normal distribution.

| **eGFR (months)** | **Early-DSA** | **Late-DSA** | **p** |
| --- | --- | --- | --- |
| eGFR 1mo* | 52.2 (±24.5) | 57.9 (±21.0) | 0.551 |
| eGFR 3mo* | 52.1 (±24.2) | 55.0 (±21.2) | 0.777 |
| eGFR 6mo* | 46.4 (±16.0) | 53.6 (±21.6) | 0.097 |
| eGFR 12mo* | 47.3 (±18.9) | 60.3 (±25.5) | 0.186 |
| eGFR 24mo* | 48.3 (±20.8) | 56.3 (±16.8) | 0.348 |
| eGFR 36mo* | 52.5 (±19.4) | 49.6 (±18.1) | 0.914 |
| eGFR 48mo* | 50.2 (±16.3) | 43.6 (±21.6) | 0.255 |

**Supplementary Figure 3. Patients with DSA and estimated glomerular filtration rate (eGFR) evolution during follow-up.**

Missing laboratory values ​​due to graft loss or lack of follow-up were imputed using last observation carried forward (LOCF) analysis.

| **DSA groups** | **eGFR** | **1mo** | **3mo** | **6mo** | **12mo** | **24mo** | **36mo** | **48mo** |
| --- | --- | --- | --- | --- | --- | --- | --- | --- |
| **Early-DSA (n=30)** | **Observed data (n)**  **LOCF (n)** | n=30  n=30 | n=30  n=30 | n=30  n=30 | n=29  n=30 | n=25  n=30 | n=19  n=30 | n=16  n=30 |
| **Late-DSA (n=19)** | **Observed data (n)**  **LOCF (n)** | n=19  n=19 | n=19  n=19 | n=19  n=19 | n=19  n=19 | n=16  n=19 | n=14  n=19 | n=13  n=19 |

**Supplementary Figure 4. Patients with DSA and time-to-eGFR reduction ≥30%.**

Time-to-eGFR decline ≥30% in patients with early and late-DSA after first DSA detection. There were no signifcant differences between patient groups (Log rank p=0.616).


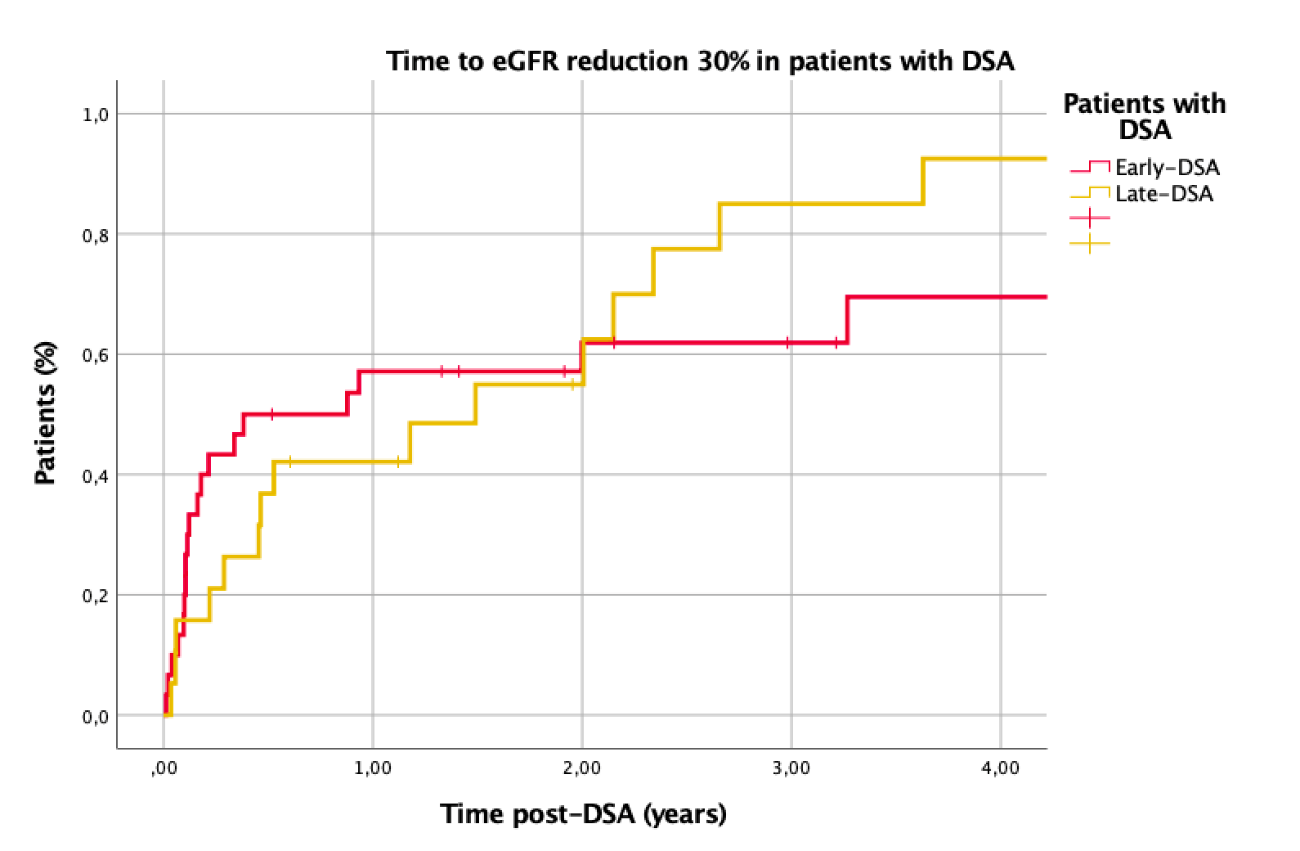


Log Rank p = 0.616

**Supplementary Figure 5. Kaplan–Meier survival analysis of death-censored graft failure for antibody-mediated rejection (ABMR) categories, independently of DSA status.**

Patients with ABMR were associated with lower allograft survival compared to patients without ABMR (log rank p=0.001). Graft survival at 5 years posttransplant was 94.2% (± 1.5%) in patients without ABMR and 84.1% (± 5.4%) in patients with ABMR.


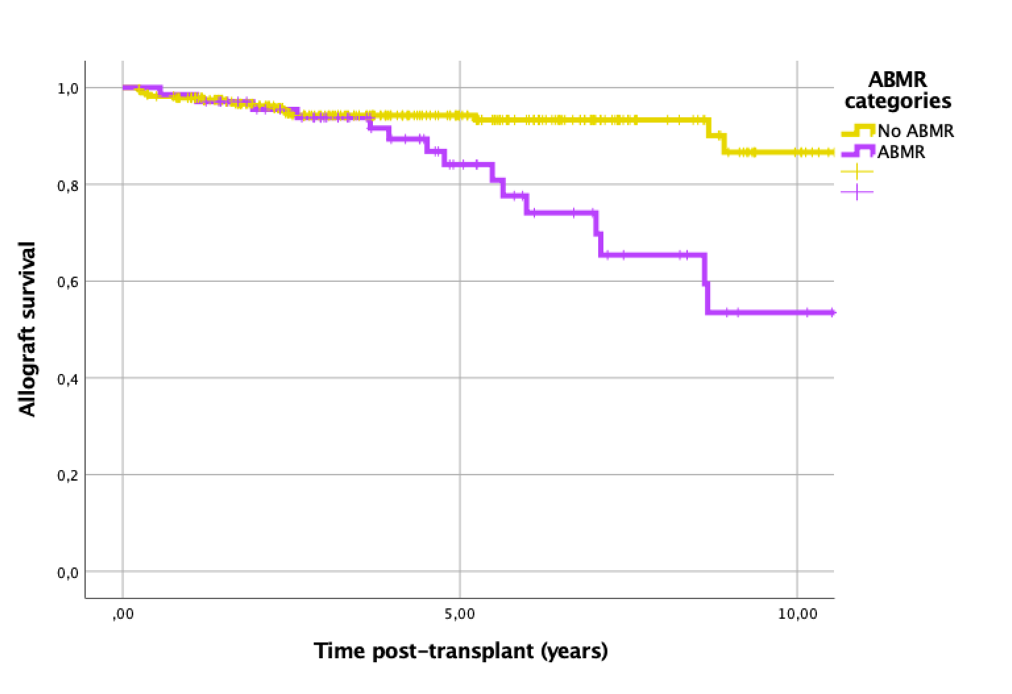


Log Rank p=0.001

**Supplementary Figure 6. Kaplan–Meier survival analysis of death-censored graft failure for T-cell mediated rejection (TCMR) categories, independently of DSA status.**

Patients with TCMR were associated with lower allograft survival compared to patients without TCMR (log rank p=0.006). Graft survival at 5 years posttransplant was 93.9% (± 1.8%) in patients without TCMR and 86.7% (± 4.0%) in patients with TCMR.

**
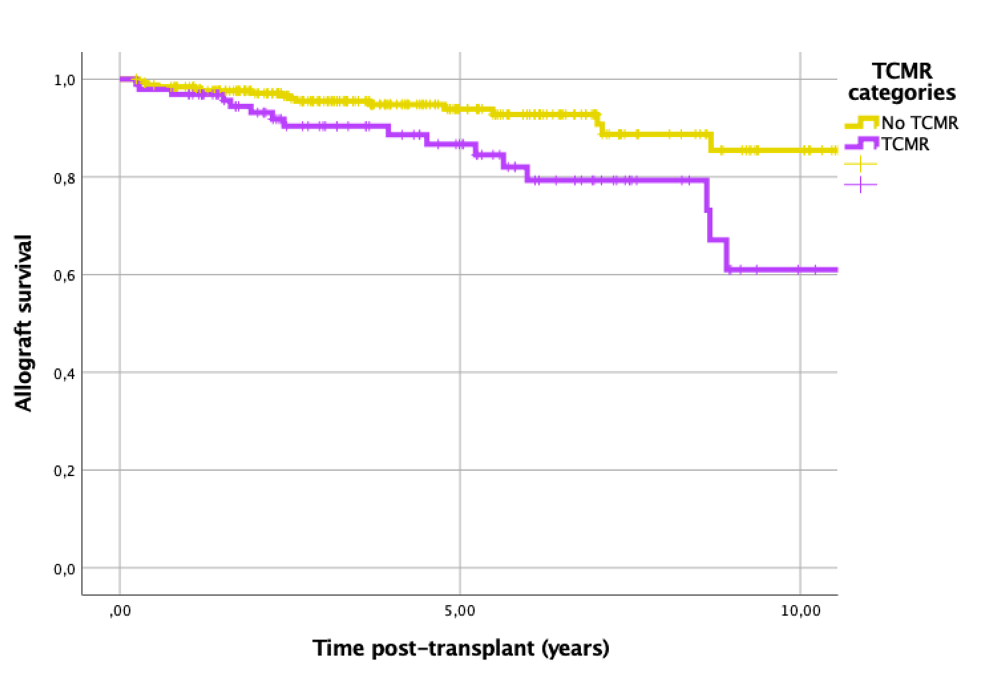
**

Log Rank p=0.006
